# Impact of Experimentally Induced Pain on Logical Reasoning and Underlying Attention-Related Psychophysiological Mechanisms

**DOI:** 10.3390/brainsci14111061

**Published:** 2024-10-25

**Authors:** Danièle Anne Gubler, Rahel Lea Zubler, Stefan Johannes Troche

**Affiliations:** Institute of Psychology, University of Bern, 3012 Bern, Switzerland; daniele.gubler@unibe.ch (D.A.G.); rahel.zubler@unibe.ch (R.L.Z.)

**Keywords:** experimentally induced pain, logical reasoning, attentional resources, upper alpha power, task-related power changes

## Abstract

Background. Pain is known to negatively impact attention, but its influence on more complex cognitive abilities, such as logical reasoning, remains inconsistent. This may be due to compensatory mechanisms (e.g., investing additional resources), which might not be detectable at the behavioral level but can be observed through psychophysiological measures. In this study, we investigated whether experimentally induced pain affects logical reasoning and underlying attentional mechanisms, using both behavioral and electroencephalographic (EEG) measures. Methods. A total of 98 female participants were divided into a pain-free control group (*N* = 47) and a pain group (*N* = 51). Both groups completed the Advanced Progressive Matrices (APM) task, with EEG recordings capturing task-related power (TRP) changes in the upper alpha frequency band (10–12 Hz). We used a mixed design where all participants completed half of the APM task in a pain-free state (control condition); the second half was completed under pain induction by the pain group but not the pain-free group (experimental condition). Results. Logical reasoning performance, as measured by APM scores and response times, declined during the experimental condition, compared to the control condition for both groups, indicating that the second part of the APM was more difficult than the first part. However, no significant differences were found between the pain and pain-free groups, suggesting that pain did not impair cognitive performance at the behavioral level. In contrast, EEG measures revealed significant differences in upper alpha band power, particularly at fronto-central sites. In the pain group, the decrease in TRP during the experimental condition was significantly smaller compared to both the control condition and the pain-free group. Conclusions. Pain did not impair task performance at the behavioral level but reduced attentional resources, as reflected by changes in upper alpha band activity. This underscores the importance of incorporating more sensitive psychophysiological measures alongside behavioral measures to better understand the impact of pain on cognitive processes.

## 1. Introduction

Pain is a multidimensional experience encompassing somatic, affective, and cognitive components [1,2]. It serves as a biologically relevant signal, alerting the body to potential harm and mobilizing physiological, cognitive, and behavioral resources to protect it against further injury. While primarily perceived through sensory experiences, pain is also processed emotionally and cognitively, thereby consuming cognitive resources [3,4]. Given the limited nature of these resources [5,6], performance on cognitive tasks in experimental or environmental settings might suffer when requiring the same resources as the processing of pain [7].

Pain has been repeatedly shown to negatively affect cognition, as evidenced by its impact on speed of information processing [8], learning and memory [9,10,11], executive functions [12,13], and especially attention [1,14,15,16]. This negative impact of pain on attention has been demonstrated in patients with chronic pain [16,17,18], in healthy individuals with naturally occurring pain [19,20], as well as in healthy individuals experiencing experimentally induced pain [18,21].

However, despite many studies pointing to the negative impact of pain on attention, the results are only partially consistent [1,14,15]. This inconsistency suggests that the underlying mechanisms by which pain affects attention are still not fully understood and need further exploration. Various factors may moderate these effects, including the origin and type of pain [18], task complexity [22], pain stimulus characteristics (e.g., novelty, intensity, predictability, and threat value [7,23,24]), and individual differences in pain catastrophizing or fear of pain [23,25].

Regarding task complexity, tasks with higher cognitive demands have been reported to be more adversely affected by pain than those with lower cognitive demands [21,22,26,27]. For simpler tasks that require minimal attention, the available attentional capacity might be sufficient to handle both the task and the pain, making the impact of pain on performance less severe. However, with increasing attentional task demands, an individual’s attentional resources may become insufficient, leading to poorer performance [21,22,26,27]. Therefore, performance may remain unaffected by pain as long as the combined demands of the task and pain do not exceed the available attentional capacity.

It appears reasonable to suggest that more complex cognitive functioning, which relies heavily on attentional systems, would be particularly vulnerable to the effects of pain. Supporting this idea, chronic pain has been found to impair performance on tasks involving abstract thinking [28], daily decision-making [29], creative ideation [30], and to some extent, logical reasoning [31]. However, this detrimental effect of pain on complex cognitive functioning could not be observed in individuals experiencing experimentally induced pain in tasks involving abstract thinking [32], creative ideation [33], or logical reasoning [34].

One potential explanation for these diverging results is that participants in studies on chronic pain versus experimentally induced pain often differ in their age and other demographic characteristics. Additionally, individuals with chronic pain frequently experience comorbid conditions such as fatigue, anxiety, or depression [35,36], which can further draw on attentional resources and negatively impact performance. Lastly, the nature of experimentally induced pain may also play a role. This type of pain is usually more controllable and can be stopped when it becomes unbearable, making it less threatening [22,32]. As a result, participants exposed to experimentally induced pain may be able to better concentrate on the task and ignore the pain, leading to fewer cognitive impairments [34].

While most studies have not observed significant performance declines at the behavioral level in individuals experiencing experimentally induced pain, cognitive mechanisms and attentional resource allocation may still be affected. For instance, individuals in pain might enhance their focus on the task at hand, apply more inhibitory control to ignore the pain, or adopt different strategies [33]. Relying solely on behavioral observations may not be sufficient to detect these nuanced changes in cognitive mechanisms. Thus, more sensitive measures may be needed to determine the effects of pain on the cognitive mechanisms underlying task performance [37,38].

The cognitive mechanisms underlying task processing can be studied using electroencephalography (EEG). This non-invasive method provides high temporal resolution and allows the measurement of oscillatory brain activity. Observing the power of different frequency bands is a common method for examining underlying processes during various cognitive activities such as logical reasoning [39,40], learning and working memory [41], and creative ideation [42]. EEG has also been employed to examine brain activity related to pain processing [43]. Regarding creative ideation, two recent studies examined the attention-related psychophysiological mechanisms underlying creative ideation in patients with chronic pain [30] and in experimentally induced pain [33]. In both studies, upper alpha power (10–12 Hz) changes—associated with inhibiting irrelevant sensory input and allocating internal attention [42]—significantly differed between the pain groups and the matched control groups. This is particularly intriguing for the present purpose since chronic pain [30], but not experimental-induced pain, negatively affected creative ideation at the behavioral level [33].

Building on these findings, the current study aimed to elucidate the detrimental impact of pain on complex cognitive functioning by investigating its effects on logical reasoning and associated psychophysiological mechanisms as measured by the EEG. We chose to investigate logical reasoning for several reasons. First, logical reasoning heavily depends on well-functioning attentional systems [44,45,46,47], making it an ideal candidate for studying the effects of pain. Second, the inconsistencies in previous research findings between the impact of chronic pain [31] and experimentally induced pain on logical reasoning [34] highlight the need to further investigate the impact of pain in this context. Finally, the psychophysiological basis of logical reasoning is well studied [40], allowing for specifying attention-relevant EEG measures that could be influenced by pain.

In this context, the power of the upper alpha band (10–12 Hz) might be of particular relevance as it decreases from a resting state to a cognitively active state [40]. This decrease is interpreted as a reflection of the activation of attentional resources [48,49]. Thus, a stronger alpha power decrease is associated with a stronger activation of attentional resources in the corresponding brain areas [41,50]. Furthermore, compared to the lower alpha power (8–10 Hz), which reflects general attentional and motivational processes, the upper alpha power (10–12 Hz) is associated with specific task-related activities and is directly linked to the task at hand [50,51,52]. Such task-related activities of the upper alpha power have also been observed during the processing of logical reasoning tasks like Raven‘s matrices [39]. These results indicate that the upper alpha power is also a sensitive measure of the activation/allocation of attentional resources required by logical reasoning.

With the current study, we aimed to explore the effects of experimentally induced pain on logical reasoning and underlying attentional mechanisms. Specifically, we aimed to examine how pain influences cognitive processes at both the behavioral and psychophysiological levels, focusing on potential changes in task performance and the allocation of attentional resources, as measured by alterations in upper alpha band power. Previous studies did not find performance differences on more complex cognitive tasks between a group experiencing experimentally induced pain and a pain-free group [32,33,34]. Sufficient attention could probably be paid to the task at hand by the participants in the pain group so that their performance was not impaired by pain. Therefore, our first hypothesis was that there would be no significant performance differences at the behavioral level between a pain and a pain-free group nor between a pain and a pain-free state.

However, we anticipated that the pain experience would lead to distinct psychophysiological patterns. Specifically, our second hypothesis was that in the absence of pain, there would be a typical decrease in the upper alpha power from a resting state to a state where participants cognitively engage in logical reasoning, indicating pronounced cognitive activation [48,49]. For those experiencing pain, two scenarios were possible. On the one hand, these individuals might show a more pronounced decrease in alpha power relative to a pain-free group or a pain-free state, indicating that they mobilized additional cognitive resources to manage the task and the pain simultaneously, thus necessitating enhanced cortical activation to uphold performance levels [49,53]. On the other hand, considering that a decrease in upper alpha power reflects the activation of attentional resources for the current task [50,51,52], the additional cognitive load of pain processing could also lead to fewer attentional resources available for the task. This would lead to a less pronounced decrease in alpha power in the pain group compared to the pain-free group or the pain-free state. In the case that the attentional resources were reduced but still sufficient, this should not lead to impaired performance at the behavioral level in the pain group compared to the pain-free group and the pain-free state. Thus, our third (non-directional) hypothesis was that the pain group would show a decrease in upper alpha power from a resting state to a state of cognitive engagement in logical reasoning, which would be more or less pronounced when compared to the typical decrease in the absence of pain.

## 2. Materials and Methods

### 2.1. Participants

Due to findings from pain induction studies indicating that females generally report lower pain thresholds to various noxious stimuli compared to males [54,55] as well as reports of sex differences in psychophysiological measures [56], this experiment included only female participants. Further inclusion criteria for the study required participants to be right-handed. Exclusion criteria included current pregnancy, epilepsy, allergies (particularly to cayenne pepper and capsaicin), recent concussions, chronic diseases, and current use of medications. A total of 121 women initially participated in the study. However, data from 23 participants were excluded due to unanalyzable EEG signal quality (16; less than 50 evaluable segments in any of the conditions), low APM values (more than three standard deviations, *SD*, below the mean), indicating a lack of motivation to complete the task (4), or outliers (more than three *SDs*above or below the mean) in the upper alpha band (3). Consequently, the final sample consisted of 98 women with an average age of 22.9 years (*SD* = 2.6, range = 18–31). Among these, one participant had an apprenticeship as their highest educational level, 68 completed high school, and 29 completed higher education. As compensation, participants who were students could choose between receiving credit points or 50 CHF, while other participants received 50 CHF. Participants were instructed to refrain from smoking or consuming caffeinated beverages one hour before the study and from drinking alcohol 24 h before the study. All participants were informed about the study protocol and provided written informed consent before their participation. The local ethics committee approved the study protocol (Project ID 2021-04-00001).

### 2.2. Instruments

Pain intensity was assessed using a visual analog scale (VAS [57]) consisting of a horizontal line with two endpoints labeled “no pain” (0) and “worst pain imaginable” (10). Participants indicated their subjective pain levels along this scale by telling the test administrator.

Handedness was measured using the Edinburgh Handedness Inventory with 11 items [58]. Participants reported whether they considered themselves right- or left-handed and indicated their preferred hand for various one- and two-handed tasks (e.g., writing, opening a drawer). Participants were classified as right-handed if they self-identified as right-handed and preferred using their right hand for more than two-thirds of the tasks.

The mini-q was used as a screening instrument to assess reasoning abilities and compare the pain group with the pain-free group before the experiment [59]. This three-minute task requires participants to judge the correctness of 64 sentences describing different symbol constellations. The mini-q demonstrates high split-half reliability (*r_tt_* = 0.98) and convergent validity with other intelligence measures (*r* = 0.37 to 0.73; [59]).

The Advanced Progressive Matrices (APM) by Raven et al. [60] were used to measure logical reasoning with 36 progressively complex items. The APM demonstrates high split-half reliability (*r* = 0.83 to 0.87) and convergent validity with other measures of logical reasoning (*r* = 0.25 to 0.50 [60]). In the present study, the items were displayed on a Dell E228WFP 22-inch Widescreen LCD Monitor (programmed with Eprime 2.0). Each item consisted of a 3 × 3 matrix with the lower right entry missing. The entries followed one or more logical rules per row and/or column. Participants were instructed to identify this rule and to select the correct missing part of the matrix from eight response alternatives. Prior to each APM item, a white cross was presented for 10 s (reference phase). Then, the APM item was presented (stimulus presentation phase) until participants responded by pressing one of the eight answer buttons (referring to the eight response alternatives) or until 90 s had elapsed.

For the present study, the 36 items were divided into two halves. The first half comprised the 18 odd-numbered items, while the second half included the 18 even-numbered items. All participants first completed the 18 odd-numbered items. Afterward, they were randomly assigned to one of the two groups (pain-free vs. pain group, see below). Subsequently, they completed the 18 even-numbered items. Before starting the actual task, two practice trials were presented. During these practice trials, any ambiguities or questions could be addressed and clarified with the test administrator.

### 2.3. Study Design and Pain Induction Procedure

A 2 × 2 mixed design was employed. The within-subjects factor contained the condition, with all participants completing the APM task in two phases: the first half (control condition) and the second half (experimental condition) of the items. The between-subjects factor contained the group assignment, with participants divided into a pain group and a pain-free group. In the control condition, both groups solved the first half of the APM without pain induction. In the experimental condition, the pain group experienced pain while solving the second half of the APM, while the pain-free group continued without pain. This design allowed for the examination of the effect of pain between groups and within the same participants across the two conditions.

After completing demographic questions, which included screening for exclusion criteria, the Edinburgh Handedness Inventory, the mini-Q, and the first half of the APM task, participants were randomly assigned to one of the two groups by a dice roll (51 in the pain group and 47 in the pain-free group). Thermal heat stimuli were administered using a quantitative sensory testing device TCS-II (QST Lab, Strasbourg, France, https://www.qst-lab.eu (accessed on 1 September 2024). The thermal heat probe had a surface area of 4.5 cm^2^. To avoid thermal damage to the skin, pain induction was combined with topically applied capsaicin, which induces neurogenic inflammation and hyperalgesia, thereby significantly lowering the pain threshold. According to Lüke et al. [61], capsaicin application reduces the pain threshold from an average of 45.3 °C to 37 °C. The capsaicin cream was applied to the left and right forearm of the participants in the pain group approximately 2 cm above the volar wrist crease. The forearms were then covered with a wound dressing and a gauze bandage to enhance the effect of the cream, with an exposure time of 25 min. The same procedure was carried out on the pain-free group with a standard moisturizing cream to maintain the comparability of the procedure in the two groups. During this 25 min period, participants filled out various personality measures on the computer screen, which were not relevant to the purpose of this study.

After the exposure time, the pain thresholds (the temperature at which the stimulus is perceived as painful) and the tolerance thresholds (the temperature at which the pain becomes unbearable) were measured separately for each forearm of each participant. For this purpose, the participants placed their forearms on the thermal heat probe attached to a holder. The temperature of the thermal heat probe started at an initial temperature of 32 °C and increased at a rate of 1 °C per second. Using a remote control, participants indicated when they had reached their pain and tolerance threshold, whereupon the thermal stimulation stopped, and the temperature of the thermal heat probe returned to 32 °C at a rate of 170 °C/s. Each threshold was measured three times per forearm, and the average values per arm were calculated separately.

**Figure 1:**
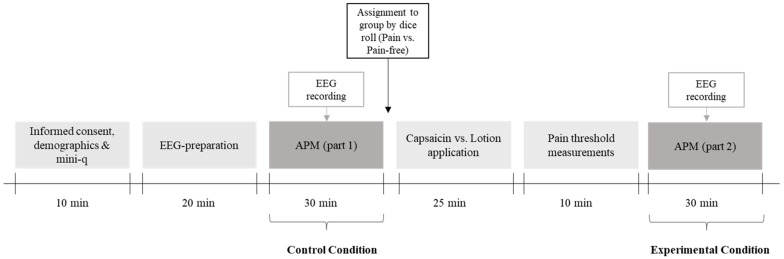
Schematic illustration of the study design. APM = Advanced Progressive Matrices.

After measuring the thresholds, participants completed the second half of the APM with thermal heat stimuli applied to the pain group during task performance. For this purpose, participants placed their forearms on the thermal heat probe before each trial. Pain induction occurred from the start of the reference phase till participants gave a response on the APM trial or after the APM trial ended after 90 s (see Figure 1). The temperature was set 1 °C above the participant’s previously determined pain threshold [61]. Pain thresholds after capsaicin application were around 39 °C, with pain induction at 40 °C resulting in an average perceived pain intensity of 4.77 (*SD* = 1.19, range = 2.7–7.0) on the VAS for participants in the pain group. As participants adapted to the heat stimuli, the thermal heat probe temperature increased by 0.5 °C every 10 s. The temperature was reset to baseline after each trial. In order to maintain an appropriate pain level, the perceived pain intensity was recorded separately for each arm after the first APM item and then after every third item, with adjustments made to the baseline temperature if necessary. No subject experienced temperatures exceeding 45 °C. Participants were allowed to remove their forearms from the probe at any time if the pain became intolerable and were explicitly instructed to do so. For reasons of comparability, the pain-free group also placed their forearms on the thermal heat probe, experiencing a pleasant heat stimulus of 34 °C throughout all APM items. Analogously to the pain group, participants in the pain-free group also reported their pain levels.

### 2.4. Electroencephalogram Recording and Analysis

For the EEG recording, a 32-channel Biosemi ActiveTwo EEG system (BioSemi, Amsterdam, The Netherlands) was employed with a sampling rate of 2048 Hz. The active gel electrodes were placed according to the international 10–20 system. EEG activity was recorded with the ActiView707 recording software. Horizontal and vertical electrooculogram (EOG) was measured with electrodes placed at the outer canthi of the right and left eyes (horizontal EOG) and with the Fp1 electrode and an electrode beneath the left eye (vertical EOG). Additionally, two flat-type active electrodes were positioned on the mastoids to serve as reference electrodes. During the recording sessions, the offset of the active electrodes was maintained below 35 μV. The common mode sense active electrode and the driven right leg passive electrode were used as the online ground.

The EEG and EOG data were analyzed using BrainVision Analyzer 2.2 software. Initially, the data were re-referenced to the average of the two mastoid electrodes. Subsequently, the EEG signal was resampled to 1024 Hz, and an offline filter (0.1 Hz) and a 50 Hz notch filter were applied. Eye movement artifacts were corrected using the Gratton and Coles [62] method, followed by visual inspection to identify and remove motion artifacts, eye blinks, and muscle tension. In five subjects, single channels exhibited poor signal quality due to pulse artifacts or muscle tension. They were replaced through interpolation using spherical splines [63].

Of particular interest to our purpose was the EEG activity during the reference phase (before a new trial was presented) and EEG activity during the stimulus presentation phase of the APM trials. From each of the 36 reference phases, segments of nine seconds duration were extracted (0.5 s after start and 0.5 s before end). Segments from the stimulus presentation phases were extracted, starting 1 s after the stimulus presentation and ending 3 s before the response was given (or the maximal item presentation was reached). These segments and those from the reference phases were further divided into 1 s segments with a 50% overlap (0.5 s) with the preceding and succeeding segments. Power estimates for all artifact-free segments were obtained using a Hanning window and subjected to a fast Fourier transformation (FFT). An average score for all segments was computed separately for the reference and stimulus presentation phases. Upper alpha power (10–12 Hz) for each participant was extracted from the FFT analysis for both phases.

Brain activity was assessed by analyzing task-related power (TRP) changes, as described by Pfurtscheller and da Silva [64]. To calculate TRP at an electrode [i], the log-transformed power during the reference phase (Pow_i_, reference) was subtracted from the log-transformed power during the stimulus presentation phase (Pow_i_, APM), using the formula: TRP = log(Powi, APM) − log(Powi, reference). Negative TRP values indicate a decrease in power from the reference to the stimulus presentation phase, while positive values indicate an increase in power. In the last step, TRP values were averaged for five brain regions: frontal (AF3, F3, F7, Fz, AF4, F4, F8), fronto-central (FC1, FC5, C3, Cz, FC2, FC6, C4), centro-parietal (CP1, CP5, P3, Pz, CP2, CP6, P4), temporal (T5, T6, T7, T8), and occipital (O1, Oz, O2).

### 2.5. Statistical Analysis

All analyses were calculated using the statistical software R version 4.3.3 and RStudio version 2024.04.2. First, differences in APM scores as well as response times to APM items between the pain and pain-free groups in both conditions (control vs. experimental) were analyzed using a two-way mixed-model analysis of variance (ANOVA) with the between-subjects factor “Group” (pain vs. pain-free group), the within-subjects factor “Condition” (control vs. experimental) and the dependent variables “APM scores” and “APM response times”.

Second, differences in TRP values were analyzed by means of five separate two-way mixed-model ANOVAs with one between-subjects factor “Group” (pain vs. pain-free group), and one within-subjects factor “Condition” (control vs. experimental) and the dependent variables “TRP values” for each position (frontal, fronto-central, centro-parietal, temporal, and occipital). Bonferroni procedure was used for post hoc pairwise comparisons. Prior to the analyses, several assumptions regarding normality, homogeneity of variance, and sphericity were tested [65]. Normality was tested using a Shapiro–Wilk normality test, homogeneity of variance using a Levene test, and sphericity using Mauchly’s test. In case that sphericity was violated, Greenhouse–Geisser correction was applied.

## 3. Results

### 3.1. Group Characteristics

First, the pain group (*N* = 51) and the pain-free group (*N* = 47) were compared according to age, educational level, and mini-q scores. There were no significant differences between the two groups in terms of age, *t*(96) = 0.261, *p* = 0.795, Cohen’s *d* = 0.05; educational level, *χ*^2^(3) = 1.264, *p* = 0.738, Cramer’s *V* = 0.11; and mini-q scores, *t*(96) = 0.338, *p* = 0.736, Cohen’s *d* = 0.07.

As depicted in Table 1, the pain and tolerance thresholds in the pain group were significantly lower than in the pain-free group, indicating that capsaicin successfully reduced these thresholds. During the APM, the pain group reported an average pain level of *M* = 4.77 on the VAS (*SD* = 1.18; *Min* = 2.70; *Max* = 7.00), which was significantly different from zero, *t*(50) = 28.880, *p* < 0.001. In contrast, the pain-free group reported no pain during the APM (*M* = 0.00, *SD* = 0.00; *Min* = 0.00; *Max* = 0.00).

### 3.2. Behavioral Results: Performance in the APM

Differences in APM scores and response times between the control and experimental conditions and between the pain and the pain-free group were tested using a two-way mixed ANOVA with APM scores and response times as the dependent variable. For APM scores, the main effect “Condition”, *F*(1, 96) = 11.571, *p* < 0.001, *η_p_*^2^ = 0.108 reached statistical significance, indicating that performance across both groups was worse in the experimental compared to the control condition. Neither the main effect “Group”, *F*(1, 96) = 0.943, *p* = 0.334, *η_p_*^2^ = 0.010, nor the interaction between “Group” and “Condition” was statistically significant, *F*(1, 96) = 0.959, *p* = 0.330, *η_p_*^2^ = 0.010. A post hoc power analysis using G*Power [66] revealed that to detect a significant interaction effect for *η_p_*^2^ = 0.010 with a power of 0.80 and an alpha error of 0.05, a sample size of 148 participants would be needed.

For APM response times, a similar pattern of results was observed for APM scores. Across both groups, participants’ response times in the control condition were significantly shorter than in the experimental condition, *F*(1, 96) = 245.097, *p* < 0.001, *η_p_*^2^ = 0.719. However, neither the main effect “Group”, *F*(1, 96) = 0.204, *p* = 0.652, *η_p_*^2^ = 0.002, nor the interaction between “Group” and “Condition” yielded statistical significance, *F*(1, 96) = 1.852, *p* = 0.177, *η_p_*^2^ = 0.019.

Means and standard deviations separately for the two groups and the two conditions, respectively, are depicted in Table 2. Across both groups, APM scores decreased significantly, *t*(97) = 3.365, *p* = 0.001, Cohen’s *d* = 0.34, and APM response times increased significantly *t*(97) = −15.656, *p* < 0.001, Cohen’s *d* = −1.58 from the control condition to the experimental condition. However, due to the non-significant main effects of “Group” and the non-significant interaction effects of “Group” × “Condition”, there were no significant differences in APM scores and response times caused by pain.

### 3.3. Psychophysiological Results: TRP Changes During APM

Differences in TRP changes between the control and experimental conditions and between the pain and the pain-free group were tested by five further two-way mixed ANOVAs with TRP changes at frontal, fronto-central, centro-parietal, temporal, and occipital electrode sites as the dependent variables, respectively. In all five ANOVAs, the interaction effect between “Condition” and “Group” was significant except for occipital electrode sites, where the interaction effect just failed to reach significance. Furthermore, the main effect “Group” was not significant in any of the five ANOVAs, while the main effect “Condition” was significant for frontal, fronto-central, and temporal, but not for the centro-parietal and occipital electrode sites.

The largest interaction effect was found for fronto-central electrode sites, *F*(1, 96) = 12.732, *p* < 0.001, *η_p_*^2^ = 0.12, so we focused on this region for the post hoc test. To ensure the robustness of this finding, a post hoc power analysis was conducted using G*Power [66]. At an alpha level of α = 0.05, a power of 1 − β = 1.00 was found for the interaction effect, indicating sufficient power to detect this effect.

**Figure 2:**
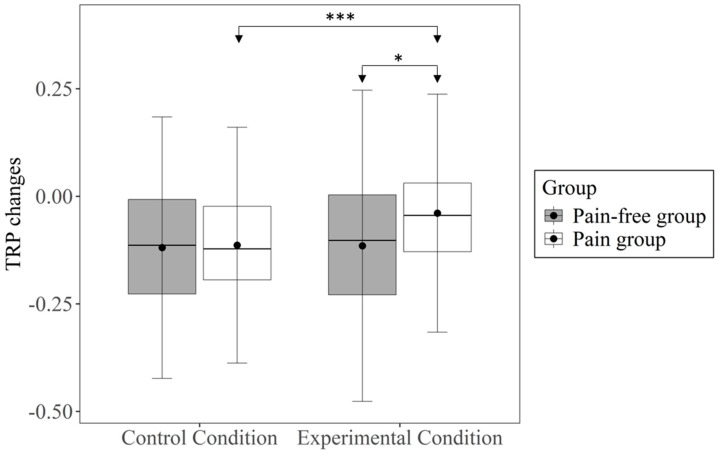
Boxplots of TRP changes in upper alpha power separated by group and condition, including means and standard deviations. TRP = task-related power. * *p* < 0.05, *** *p* < 0.001.

The four TRP values (from the two groups in the two conditions) are depicted in Table 2 and Figure 2. These values were all negative and differed significantly from zero, all *ts*(50) < −2.027, and all *ps* < 0.048, indicating that across all groups and conditions, the upper alpha power decreased from the resting state to the state of logical reasoning. In the control condition, TRP values did not differ between the pain group (which did not experience pain in this condition) and the pain-free group, *t*(956) = −0.198, *p* = 0.843, Cohen’s *d* = −0.04. In the experimental condition, however, the TRP values of the pain group were significantly less pronounced (i.e., less negative) than the TRP values of the pain-free group, *t*(96) = −2.340, *p* = 0.021, Cohen’s *d* = −0.47.

Furthermore, in the pain group, TRP decreases were significantly less pronounced (i.e., less negative) in the experimental condition compared to the control condition, *t*(50) = −5.210, *p* < 0.001, Cohen’s *d* = −0.73. In the pain-free group, TRP values did not differ between the control condition and the experimental condition, *t*(46) = −0.329, *p* = 0.774, Cohen’s *d* = −0.05. These results indicate that pain induction significantly reduced TRP when comparing a pain group and a pain-free group, as well as when comparing a pain state and a pain-free state within the same individuals.

## 4. Discussion

In this study, we explored how experimentally induced pain affects logical reasoning and underlying psychophysiological attentional mechanisms. Our findings demonstrated no significant differences in a logical reasoning task between a pain group and a pain-free group at the behavioral level, so our first hypothesis did not have to be rejected. However, we observed notable differences at the psychophysiological level, particularly at fronto-central electrode sites. Consistent with our second hypothesis, the pain-free group showed a pronounced TRP decrease from a resting state to a cognitively active state, where participants completed the APM items. In line with our third hypothesis, this decrease in upper alpha power was significantly less pronounced in the pain group compared to the pain-free group. Additionally, within individuals, the decrease in upper alpha power was significantly less pronounced during the pain condition compared to the pain-free condition.

The behavioral results revealed that experimentally induced pain did not negatively affect logical reasoning performance. This finding aligns with previous research in which no significant differences could be found between individuals experiencing experimentally induced pain and those who were pain-free in more complex cognitive abilities such as logical reasoning [34], creative ideation [33], and abstract thinking [32]. One possible explanation for this finding could be that the simultaneous processing of the task and the pain may not have fully exhausted the cognitive resources of the participants experiencing pain [26]. Consequently, both groups performed similarly well on the task, although the pain group may have experienced a higher attentional demand that was not reflected in their behavior.

Several reasons may explain why the cognitive resources of individuals in pain were not fully depleted. First, the characteristics of experimentally induced pain (e.g., duration, predictability, and threat value [7,23,67]) might have contributed to this result. Specifically, in this study, the pain stimulus was only applied over a short duration and only occurred within the trials, which made it more predictable. Participants also knew that the heat from the thermal heat probe must not exceed a predetermined temperature of 45 °C, and they were explicitly instructed that they could escape the pain at any time. Consequently, this made the pain stimulus less threatening compared to chronic pain or naturally occurring pain, thereby possibly reducing its distracting influence on logical reasoning.

Furthermore, the task may have been sufficiently engaging to hold participants’ attention away from the pain, allowing them to prioritize the task over the pain experience [68]. This result would also be consistent with studies that have shown that a cognitively demanding task leads to reduced pain experience, suggesting that attention can be diverted from pain to the task as long as the pain is not threatening enough [22,24,26].

Lastly, participants who experienced experimentally induced pain may have been more motivated to perform well in the second half of the APM task than participants without pain who simply repeated the same task with different items. Van Damme et al. [68] argue that motivation plays a crucial role in how pain affects attention, with higher motivation helping individuals to cope with pain and maintain performance. This is consistent with studies showing that motivation can improve task performance under pain [69,70]. This increased motivation in the pain group could, therefore, explain the lack of performance differences, as it likely compensates for pain-related attentional deficits.

To investigate the extent to which underlying cognitive mechanisms were affected by pain, we further examined the impact at the psychophysiological level. In line with our assumption, upper alpha power decreased significantly from a resting state to a state where participants engaged in logical reasoning. Considering upper alpha power decreases as an indication of cognitive activation [48,49], logical reasoning required substantial attentional engagement in the present study. In the pain-free group, this decrease in upper alpha power did not differ significantly between the two conditions in which the two halves of the APM were solved, suggesting that both conditions required approximately the same level of attentional engagement. However, participants in the pain group exhibited a significantly less pronounced decrease in upper alpha power during the experimental condition in which they experienced pain compared to the control group. Thus, the pain group activated less attentional resources during the logical reasoning, maybe because more attentional resources were not available due to the concurrent processing of the pain. The reduced attentional resources in the pain group were observed not only in comparison to the control group but also within the pain group between the control and experimental conditions. This emphasizes that the experience of acute pain probably consumed some of the attentional resources, leaving a smaller amount for processing additional tasks such as logical reasoning in individuals affected by pain.

When combining the behavioral and psychophysiological results, it becomes evident that the reduction in attentional resources due to pain, as visible in the EEG, did not lead to performance losses at the behavioral level. This suggests that, although the pain likely drained some attentional resources, the remaining cognitive resources were still sufficient for participants with pain to perform the logical reasoning tasks as well as those without pain. However, adding psychophysiological measures provided deeper insights into how pain affects underlying cognitive mechanisms sensitive to task processing, insights that cannot be revealed by behavioral measures alone [37,38].

An interesting area of research would be determining the point at which the simultaneous processing of pain and a task exhausts attentional resources so strongly that behavioral performance declines. In addition, it would be interesting to investigate how alpha power is affected during a logical reasoning task in individuals suffering from chronic pain. Previous research has already demonstrated that chronic pain impairs performance on more complex cognitive tasks at the behavioral level [29,30,31]. However, the extent to which attentional resources are constrained during the processing of complex cognitive tasks in chronic pain, as measured at the psychophysiological level, remains largely unexplored.

Despite reduced attentional resources at the psychophysiological level, the lack of behavioral differences could further indicate that individuals experiencing pain were employing compensatory strategies to maintain their performance. For example, they might be using additional cognitive resources to actively suppress the pain while focusing on the task or switching their attention between the pain and the task without a noticeable decline in performance. This would be in line with studies demonstrating that individuals with better inhibitory abilities performed better in a cognitive task under pain [71,72]. These compensatory strategies might not be reflected in the (task-specific) upper alpha band of the EEG but could involve different frequency bands and interactions between these frequency bands or between various brain regions. Exploring these dynamics could provide deeper insights into how pain influences cognitive functioning and how individuals adapt to maintain performance under pain.

Furthermore, the various processes involved in logical reasoning could also be examined in more detail. For example, solving a logical reasoning task consists of several cognitive steps that need to be performed [72]. Instead of averaging the upper alpha activity over the entire task period, these specific processes could be examined individually. In this way, specific stages at which pain leads to differences in the underlying cognitive processes could be identified.

### Limitations

There are several limitations to this study that affect the generalizability of its findings. As previously mentioned, experimentally induced pain may not fully capture the complexity of real-life acute pain conditions, such as headaches, back pain, or abdominal pain, which often involve greater levels of threat, unpredictability, and intensity. Consequently, the results of this study should not be generalized to other types of acute pain but rather interpreted as the effects of experimentally induced pain on cognitive mechanisms. Nonetheless, the fact that even this “mild” form of pain produced detectable impairments, particularly at the psychophysiological level, suggests that more severe or naturally occurring pain conditions could lead to more pronounced effects. Furthermore, the applicability of these findings to chronic pain is even more limited, given the fundamental differences in pain characteristics and the common comorbidities associated with chronic pain patients [35,36]. Chronic pain is often accompanied by long-term changes in brain structure [1,73], which are likely to exacerbate cognitive impairments beyond those observed in this study.

A further limitation includes the homogeneous study sample, which consisted only of females, primarily university students with a mean age of 22.9 years. This restricts the generalizability of the findings, as there is evidence that pain perception and experience can vary by gender [74,75] and age [76]. Thus, comparisons to more diverse populations, such as middle-aged individuals or those with varying educational backgrounds, remain challenging. Future research should explore whether similar effects of pain on cognitive mechanisms are observed across different demographic groups.

Lastly, a post hoc power analysis indicated that with a larger sample size, it is likely that a significant interaction effect at the behavioral level would have been detected. This suggests that the current sample size may have been too small to capture these effects. However, the observed effect size was small, and the practical significance of such a small effect remains uncertain.

## 5. Conclusions

In conclusion, the present study demonstrates that experimentally induced pain does not impair logical reasoning performance at the behavioral level as participants in the pain group performed comparably to those in the pain-free group. However, at a more sensitive psychophysiological level, attentional resources were likely reduced, as shown by the less pronounced decrease in upper alpha power in the pain group compared to the pain-free group and in a pain state compared to a pain-free state. This suggests that while performance was maintained, pain still affected the allocation of attentional resources. These findings highlight that even experimentally induced pain can influence cognitive processes when measured at a more sensitive level. Future research is needed to explore the thresholds at which pain and task complexity lead to the depletion of attentional resources or overstrain compensatory mechanisms, resulting in a decline in performance. Additionally, further research is needed to better understand the differences between chronic, naturally occurring, and experimentally induced pain, particularly in terms of qualitative differences in the pain experience.

## Figures and Tables

**Table 1:** Pain and tolerance thresholds for the pain-free group and the pain group after capsaicin application.

	Pain-Free Group (*N* = 47)		Pain Group (*N* = 51)			
	Mean	*SD*	Mean	*SD*	*t* test (df = 96)	Cohen’s *d*
Pain threshold (left arm)	43.09	3.99	39.70	4.15	4.112 ***	0.831
Pain threshold (right arm)	41.97	4.12	38.65	4.47	3.793 ***	0.771
Tolerance threshold (left arm)	47.46	2.22	45.07	3.57	3.936 ***	0.796
Tolerance threshold (right arm)	46.86	2.77	43.90	4.32	3.989 ***	0.807

Note. *SD* = Standard Deviation. *** *p* < 0.001

**Table 2:** APM scores and response times in the control condition (first half of APM items) and the experimental condition (second half of APM items), separated for the pain-free group and the pain group.

	Pain-Free Group (*N* = 47)				Pain Group (*N* = 51)			
	Control Condition		Experimental Condition		Control Condition		Experimental Condition	
	Mean	*SD*	Mean	*SD*	Mean	*SD*	Mean	*SD*
APM Sum Scores (0–18)	11.94	2.22	10.98	2.46	11.31	2.33	10.78	2.36
APM Response Times (0–90 s)	35.27	9.29	44.76	10.30	33.51	9.12	44.81	10.69
TRP changes at fronto-central electrode sites	−0.119	0.15	−0.115	0.18	−0.114	0.14	−0.039	0.14

Note. APM = Advanced Progressive Matrices, TRP = task-related power, *SD* = Standard Deviation.

## Data Availability

Data and the analysis script are publicly available at the Open Science Framework and can be accessed at https://osf.io/xc5sr (accessed on 23 October 2024).
